# Ceramide-containing liposomes with doxorubicin: time and cell-dependent effect of C6 and C12 ceramide

**DOI:** 10.18632/oncotarget.20217

**Published:** 2017-08-12

**Authors:** Anders Øverbye, Ann Mari Holsæter, Fusser Markus, Nataša Škalko-Basnet, Tore-Geir Iversen, Maria Lyngaas Torgersen, Tonje Sønstevold, Olav Engebraaten, Kjersti Flatmark, Gunhild Mari Mælandsmo, Tore Skotland, Kirsten Sandvig

**Affiliations:** ^1^ Centre for Cancer Biomedicine, Faculty Division Norwegian Radium Hospital, University of Oslo, Oslo, Norway; ^2^ Department of Molecular Cell Biology, Institute for Cancer Research, The Norwegian Radium Hospital, Oslo University Hospital, Oslo, Norway; ^3^ Drug Transport and Delivery Research Group, Department of Pharmacy, Faculty of Health Sciences, University of Tromsø – The Arctic University of Norway, Tromsø, Norway; ^4^ Department of Tumour Biology, Institute for Cancer Research, The Norwegian Radium Hospital, Oslo University Hospital, Oslo, Norway; ^5^ Institute for Clinical Medicine, Faculty of Medicine, University of Oslo, Oslo, Norway; ^6^ Department of Biosciences, University of Oslo, Oslo, Norway

**Keywords:** liposomes, ceramide, doxorubicin, mice tumors, cell studies

## Abstract

Doxorubicin, a widely used chemotherapeutic drug, has several potential high-risk side effects including cardiomyopathy. Furthermore, cellular resistance to this drug develops with time. By using liposomes as carrier vesicles both the side effects and drug resistance might be avoided. In this study we have investigated the cytotoxic effect of doxorubicin encapsulated in liposomes with and without ceramides containing 6 or 12 carbon atoms in the N-amidated fatty acyl chains. The short-chain ceramide species were included in the liposomal compositions due to their pro-apoptotic properties, which might cause a synergistic anticancer effect. We demonstrate that the ceramide species enhance the liposomal doxorubicin toxicity in a cell-specific manner. The C6-ceramide effect is most pronounced in cervical cancer cells (HeLa) and colon cancer cells (HCT116), whereas the C12-ceramide effect is strongest in breast cancer cells (MDA-MB-231). Moreover, the study reveals the importance of investigating cell toxicity at several time points and in different cell-lines, to assess drug-and formulation-induced cytotoxic effects *in vitro*. Furthermore, our data show that the cytotoxicity obtained with the nanocarriers *in vitro*, does not necessarily reflect their ability to inhibit tumor growth *in vivo*. We speculate that the larger effect of Caelyx® than our liposomes *in vivo* is due to a greater *in vivo* stability of Caelyx®.

## INTRODUCTION

Chemotherapy has improved the prognosis of a number of different cancer diagnoses during the last decades, but unwanted drug effects on healthy tissue remain a major problem. A challenge is the heterogenic nature of the tumor and the microenvironment, as well as the inherent or acquired drug resistance of tumor cells, in addition to the adverse effects seen on normal tissue. An emerging approach to overcome these challenges is to use drug-loaded nanocarriers that may maximize tumor growth inhibition, reduce the systemic toxicity, and overcome drug resistance.

Liposomes are suitable drug carrier systems for therapeutic applications in cancer treatment, and able to incorporate drugs with different physicochemical properties [1, 2]. The properties of liposomes depend on the lipid composition, as it will affect the biodistribution, surface charge, drug permeability and drug release, as well as clearance of the liposomal drug from the body. Thus, liposomes may protect the drug from enzymatic degradation, improve the drug pharmacokinetics, and tissue distribution, and provide a sustained or controlled release of therapeutic agents at appropriate sites [3, 4].

One of the most successful liposome-encapsulated drugs is doxorubicin (DOX), available as the marketed (PEGylated liposomal) product Doxil®/Caelyx® [5, 6], approved by FDA in 1995. Its prolonged circulation time and gradual release of DOX led to increased efficacy and reduced cardiomyopathy. However, higher susceptibility towards hand-and-foot disease (*palmar plantar erythrodysthesia*) in patients treated with Doxil®/Caelyx® [7] drives the investigations towards improved formulations of PEGylated liposomal DOX.

Incorporation of ceramide into the liposomal lipid bilayer could improve the therapeutic effect of liposomes [8]. Ceramide, a key molecule in sphingolipid metabolism, is composed of a sphingosine base and an amide-linked acyl chain varying in length; the endogenous species most commonly contain fatty acyl groups with 16 to 24 carbon atoms. It is a bioactive sphingolipid linked to induction of senescence, growth inhibition, and death in cancer cells [9, 10]. The PI3K-PKB/Akt signalling cascade determines much of a cell's growth potential, and is strongly linked to tumorigenesis. This pathway is considered the main route for cellular effects of ceramide, and downstream targets include several stress-activated protein kinases [11]. Additionally, ceramide potently inhibits angiogenesis through IFN-gamma activation [12]. Cellular membranes with different ceramide species, with varying hydrocarbon chain lengths, also show dissimilar properties in terms of stability and rigidity [13–15]. This suggests that ceramide modulation of liposomal membranes would impact intracellular transport of the liposomes [16]. The free form of the short-chain ceramide C6 has been shown to be an effective mediator of caspase-dependent cell death, but is surpassed by a similar dose of liposomes containing this ceramide species [17], due to low solubility in cell medium and less efficient cellular uptake of the free ceramide C6. A change in liposomal structure could also be beneficiary to increase cellular permeability [18].

DOX is an anthracycline antibiotic with antineoplastic activity. The drug exerts its cytotoxicityby intercalating with DNA base pairs, as well as interacting with several molecular targets such as DNA topoisomerase II to produce a range of effects [19]. Importantly, it is also reported to increase the intracellular ceramide level, further enhancing the apoptotic potential [20]. The resistance to DOX seen in several cancer types is partly attributed to the induced activity of glucosylceramide synthase (GCS) [15, 21, 22]. GCS glucosylates ceramide and thereby counteracts the pro-apoptotic effects of ceramide. By adding exogenous ceramides concomitantly with doxorubicin one might reduce GCS-mediated depletion of pro-apoptotic ceramide [23].

The size of liposomes and other nanoparticles has a direct effect on their circulation time and biodistribution following intravenous injection. Particles with a diameter below 200 nm are taken up into liver and spleen (the reticuloendothelial system) more slowly than larger particles, and thus relatively more of the injected dose is being trapped in tumors due to the enhanced permeability and retention (EPR) effect [24]. In the present study we have investigated the effect of ceramide with different chain lengths (C6 and C12) in PEGylated liposomes with encapsulated DOX. The effect of such liposomes was studied in various cancer cell lines and in a mice breast cancer model, i.e. the orthotopic basal-like xenograft model MAS98.12.

## RESULTS

### Liposome characterization

**Figure 1:**
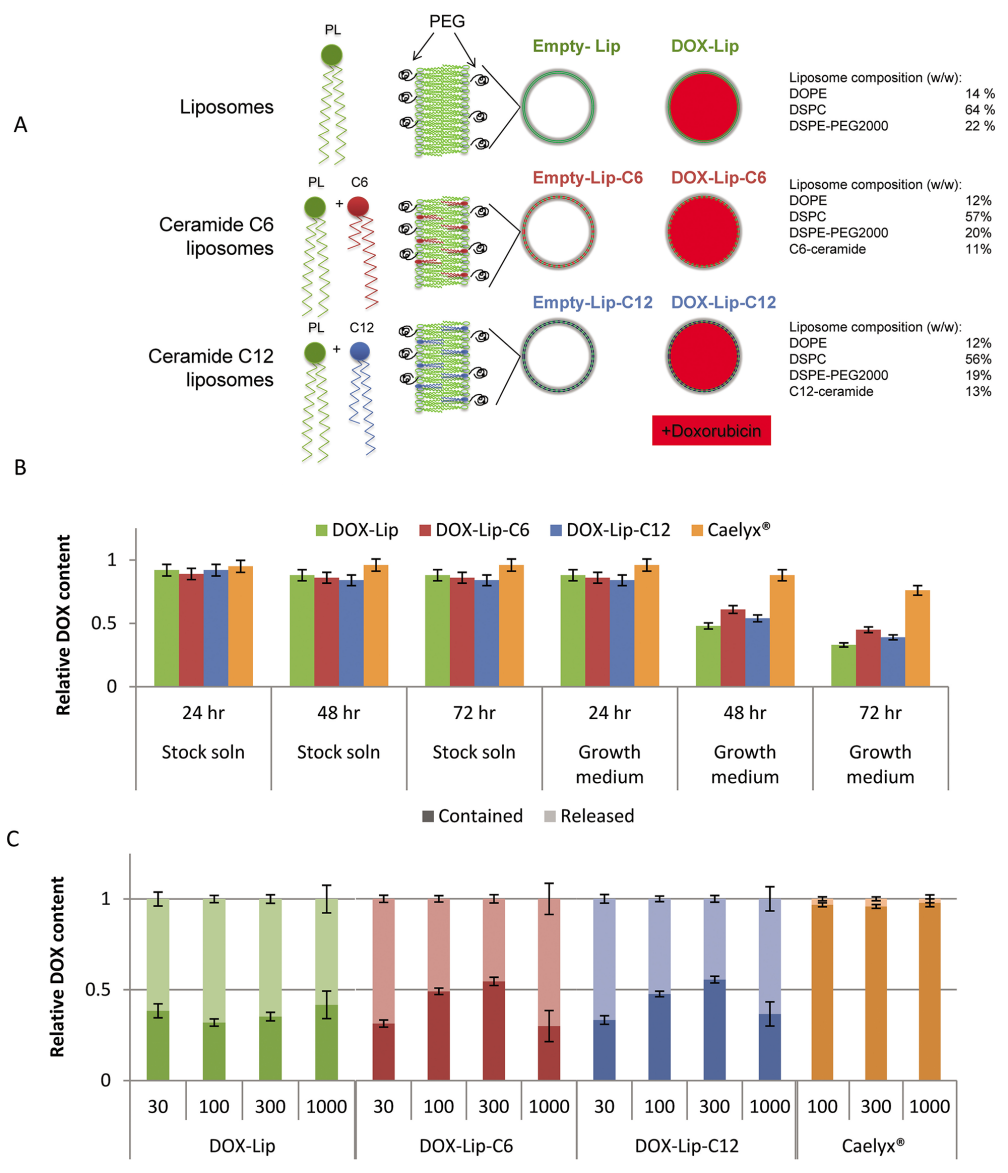
Description of liposomal formulations used in the study **(A)** Illustrations of liposomes used. PL= phospholipids, PEG = polyethylene glycol, DOPE = dioleoylphosphatidyl-ethanolamine, DSPC = distearoyl-sn-glycero-3-phosphocholine, DSPE = 1,2-distearoylphosphatidyl-ethanolamine. **(B)** Liposomal stability in solution. DOX-containing liposomes (1 mM) were incubated in stock solution (5% (w/v) sucrose solution pH 5.0 or in growth medium (DMEM with 10% (v/v) FCS, pH 7.4 for 24, 48 or 72 h. The liposomes were filtered and DOX was quantified to determine the percent of DOX retained in liposomes. The values show average of three experiments with standard deviations. **(C)** Effect of ceramide on doxorubicin release from liposomes. HeLa cells were incubated with various concentrations (30 – 1000 nM) of four different DOX-loaded liposomes and Free-DOX. After 24 h the cell medium was removed, cells washed and lysed using 0.1% (v/v) Triton-X100. DOX still encapsulated in liposomes was separated from free/released DOX by solid phase extraction and quantified. The data show the percent of DOX being encapsulated in liposomes or free. Mean values obtained by analyzing 3 replicates are shown.

The size and polydispersity index (PDI) of our liposomal formulations (Figure 1A) were compared to the average size and PDI of the marketed PEGylated liposomal product Caelyx®. All our liposomal formulations exhibited larger PDI than our measurements of Caelyx® (Supplementary Table 1). However, the PDI of our liposomal formulations was comparable to that reported in other studies with ceramide-containing liposomes [25]. Inclusion of ceramide equimolar to doxorubicin into liposomes did not affect their physical characteristics. Figure 1B displays the retention of DOX in the different liposome formulations, i.e. DOX-Lip, DOX-Lip-C6, DOX-Lip-C12 and Caelyx®, after being dispersed in two different solutes; 5% (w/v) sucrose solution (pH 5.0), and cell growth medium (DMEM+FCS, pH 7.4), respectively. The amount of DOX released from the liposomes at various time points was determined by HPLC. The stability of all liposomal formulations was higher in storage solution than in growth medium. A higher DOX-release was observed from the investigated formulations than for Caelyx® when these formulations were incubated in the medium (Figure 1B). The DOX-containing liposomes exhibited a half-life of 40-55 h for the release of DOX, whereas a small increase in half-life, although not significant, was observed for the ceramide-containing liposomes.

The characteristics of the liposomal formulation in cell cultures were also determined. HeLa cells were incubated with the different DOX-containing liposomes for 24 h. To separate encapsulated from released DOX, mild lysis conditions (0.1% (v/v) Triton X-100) were used to lyse the cells, but not the liposomes (Supplementary Figure 1). The lysate was separated from intact liposomes on a solid phase extraction C18 column to distinguish between liposomal and unbound DOX, and the DOX content in each fraction was measured relative to the same concentration of untreated vesicles/compound after extraction with 1.5% (v/v) Triton X-100 (Figure 1C). The resulting data show that release of DOX is concentration-dependent and that for all liposomal formulations, beside Caelyx®, only between 5 and 20% is extractable after 1 h (Supplementary Figure 2), which increases to 45-60% after 3 h, and reaches 70-80% after 24 h. For Caelyx® only a very minor fraction of the total DOX is not contained in the liposomal form even after 24 h.

### Liposomal ceramides induce higher toxicity for DOX in cells

**Figure 2:**
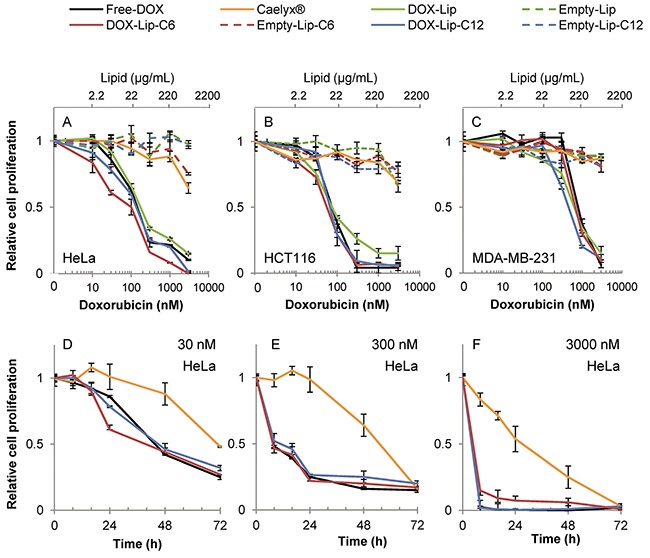
Dose-dependent effect of liposomal doxorubicin on cell proliferation **(A-C)** Three different cell lines were incubated for 24 h with various concentrations of liposomal DOX (10-3000 nM) followed by measuring incorporation of [^3^H]thymidine. Free-DOX, empty liposomes with same lipid concentration or no treatment was used for comparison. A) HeLa, B) HCT116, C) MDA-MB-231. **(D-F)** Time-dependent effect of liposomal DOX on the cell proliferation. HeLa cells incubated with increasing liposomal DOX-concentrations (30, 300 and 3000 nM) or empty liposomes with same lipid concentration were incubated for up to 72 h and the cell proliferation was determined by measuring the incorporation of [^3^H]thymidine (Free-DOX used for comparison) at various time points (8 - 16 - 24 - 48 - 72 h). D) HeLa 30 nM, E) HeLa 300 nM, F) HeLa 3000 nM. The data show the mean values from at least three independent experiments and standard deviations. The total lipid concentration of the formulations is displayed on the secondary x-axis in A-C.

The cytotoxicities obtained with the different preparations measured as cell proliferation with [^3^H]thymidine incorporation, are shown in Figure 2 and the IC50-values are shown in Supplementary Table 2. The results indicate a significantly higher toxicity of ceramide C6-containing liposomes loaded with doxorubicin (DOX-Lip-C6), as compared to free doxorubicin (Free-DOX) in HeLa (Figure 2A) and HCT116 (Figure 2B) cells after 24h of incubation. The effect is especially prominent at lower concentrations (below 100 nM). In contrast, the breast cancer cell line MDA-MB-231 (Figure 2C) exhibited a higher sensitivity towards DOX-Lip-C12 liposomes than DOX-Lip-C6, and was, in general, more resistant to DOX than the other cell lines. Ceramide liposomes without DOX and Caelyx® were much less toxic to these cells after 24 h incubation, although some minor toxic effects were observed at the highest concentration of 3 μM.

Figure 2D-2F displays the time/concentration-dependent relationship between different formulations during 72 h incubation of HeLa cells, in particular revealing the delayed effect of Caelyx®. These results demonstrate the sustained release of DOX from liposomes when compared to Free-DOX (see also Supplementary Figure 3A). The delayed toxic effect was most prominent at low concentrations (Figure 2D; 30 nM), but it is evident for Caelyx® also at higher concentrations. In summary, it appears that different ceramide chain lengths (C6 for HCT116 and HeLa; C12 for MDA-MB-231) exhibit synergistic cell toxicity with DOX; the synergy being most prominent at low dosage, and thus the combined ceramide and DOX treatment might allow use of lower concentrations of DOX in cancer treatment, retaining the same efficacy.

**Figure 3:**
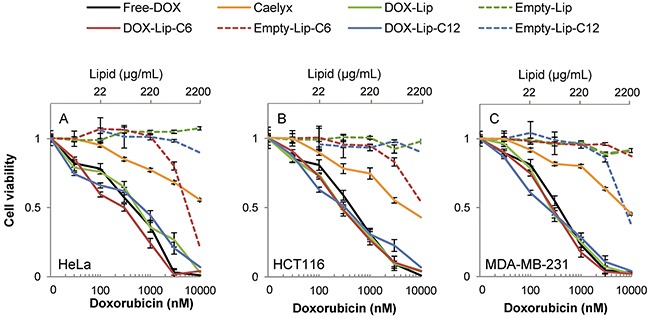
Dose-dependent effect of liposomal doxorubicin on cell viability Cells were incubated for 72 h with various concentrations of DOX-containing (30 - 3000 nM DOX) liposomes and compared to Free-DOX and empty liposomes with the same lipid concentration. Cell viability was determined by using the MTT assay. **(A)** HeLa, **(B)** HCT116, **(C)** MDA-MB-231. The data show the mean values from at least three independent experiments and standard deviations. The total lipid concentration of the formulations is displayed on the secondary x-axis.

To assess whether ceramide alone influences cell survival more efficiently over time, different concentrations of empty liposomes were tested in the same three cell lines using the MTT assay, since this viability assay addresses the integrity of the mitochondrial metabolic activity – a known target for ceramide toxicity [26, 27]. High concentrations of ceramide C6 (>3 μM) were highly toxic to HeLa (Figure 3A) and HCT116 cells (Figure 3B) after 72 h, and in HeLa cells the toxicity was already prominent after 24 h (Supplementary Figure 3B). The MDA-MB-231 cells, tested in the same assay (Figure 3C), exhibited higher sensitivity towards ceramide C12 than the other cell lines, which remain mostly unaffected by the exposure to empty C12 liposomes.

The IC_50_-values observed for DOX-treatments in MDA-MB-231 cells for the MTT assay compared to the other two cell lines are comparable, in contrast to the difference seen for cell proliferation measured by [^3^H]thymidine incorporation between the cell types (summarized in Supplementary Table 2). This reflects either that the MDA-MB-231 cell line has a more robust cell division, or a larger dependency on mitochondrial activity, or both. Another option is the difference in plasma membrane composition among the cell lines.

### Higher amounts of ceramide affect DOX toxicity in a cell-dependent manner

**Figure 4:**
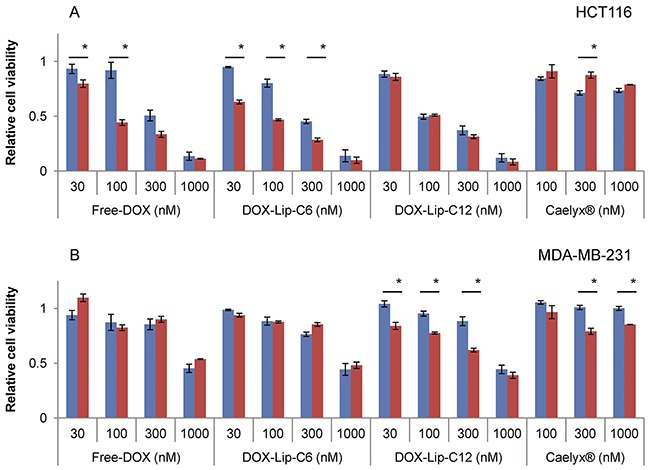
Dose-dependent effect of increased ceramide addition to liposomal doxorubicin on cell viability **(A)** HCT116, **(B)** MDA-MB-231. Cells were incubated with various concentrations (30 nM - 1000 nM) of DOX-containing liposomes for 72 h. One part of the samples had an additional 1 μM of ceramide of same type of ceramide-liposomes without DOX added. Empty-Lip-C6 were added to DOX-Lip-C6 and Free-DOX, and Empty-Lip-C12 were added to DOX-Lip-C12 and Caelyx® and incubated for 72 h. The comparison was made between no addition (blue bars) and additional ceramide-containing liposomes (red bars). The cells’ viability was determined by using the MTT assay. The data show the mean values from three independent experiments and standard deviations. *p<0.05.

To investigate the relationship between the cytotoxicity of ceramide and DOX, cells were treated with DOX-loaded liposomes giving a DOX and ceramide concentration between 0.03 and 1 μM. Half of the samples were further treated with increased amounts of ceramide by adding Empty-Lip-C6 to cells treated DOX-Lip-C6, or Empty-Lip-C12 to cells treated with DOX-Lip-C12 equivalent to an additional 1 μM ceramide. The same additions of Empty-Lip-C6 to Free-DOX and of Empty-Lip-C12 to Caelyx® are shown for comparison. The cytotoxicity was determined by the MTT cell viability test after 72 h (Figure 4A-4B). Ceramide addition was most potent when combined with Free-DOX, but also significantly increased the toxicity of DOX-Lip-C6 at lower concentrations in the HCT116 cell line (Figure 4A). In contrast, the MDA-MB-231 cells were more sensitive to additional amounts of ceramide C12 (Figure 4B). The toxic effect of Caelyx® was also slightly affected by addition of C12 ceramide, which has a protective role in HCT116, but sensitizing in MDA-MB-231. Similar effects were observed when adding Empty-Lip-C12 together with Free-DOX or Empty-Lip-C6 together with Caelyx® (data not shown).

### Influence of ceramide in liposomes on kinetics of DOX-release

**Figure 5:**
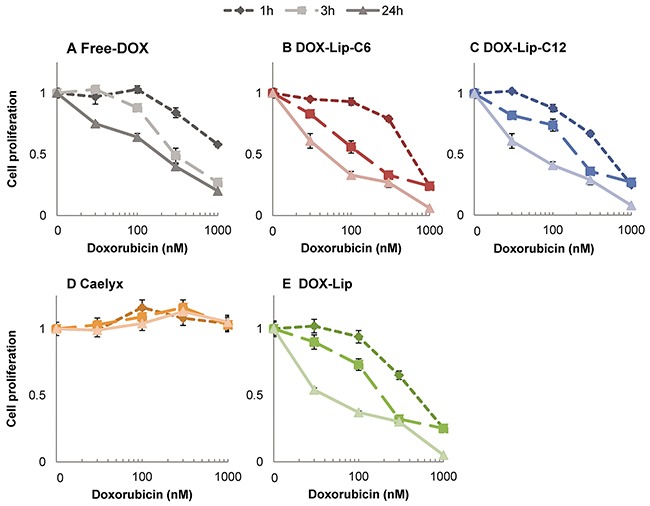
Influence of ceramide in liposomes on kinetics of DOX effect on cell proliferation **(A)** Free-DOX, **(B)** DOX-Lip-C6, **(C)** DOX-Lip-C12, **(D)** Caelyx®, **(E)** DOX-Lip. HeLa cells were incubated with various concentrations (30 – 1000 nM) of four different DOX-loaded liposomes and Free-DOX. After 1 and 3 h, one third of the cells were washed twice, fresh medium was added and the incubation extended. After a total of 24 h incubation the cell proliferation were compared with that of continuously treated cells. The data show the mean values from three independent experiments and standard deviations.

In order to study in more detail the kinetics of the intoxication with the liposome formulations, HeLa cells were incubated for 1, 3 and 24 h with the DOX containing liposomes (DOX-Lip-C6, DOX-Lip-C-12, DOX-Lip and Caelyx®) and Free-DOX. All cells were grown for 24 h, but for 1 and 3 h incubation samples, cells were washed (twice) and medium changed after 1 and 3 h, respectively, prior to continued incubation for a total of 24 h. At the end of the incubation, the cell proliferation was determined by the [^3^H]thymidine incorporation assay (Figure 5). A slightly more prominent cell toxicity was observed for the DOX-Lip-C6 treated cells, as compared to cells treated with ceramide-free formulations (DOX-Lip) after 3 h (p>0.05), but in general only minor differences were observed. This lack of impact of ceramide on the kinetics of drug release was also seen when quantifying DOX retained and released from liposomes given to cells (Supplementary Figure 2.).

### Ceramide and DOX affect different cell death mechanisms

**Figure 6:**
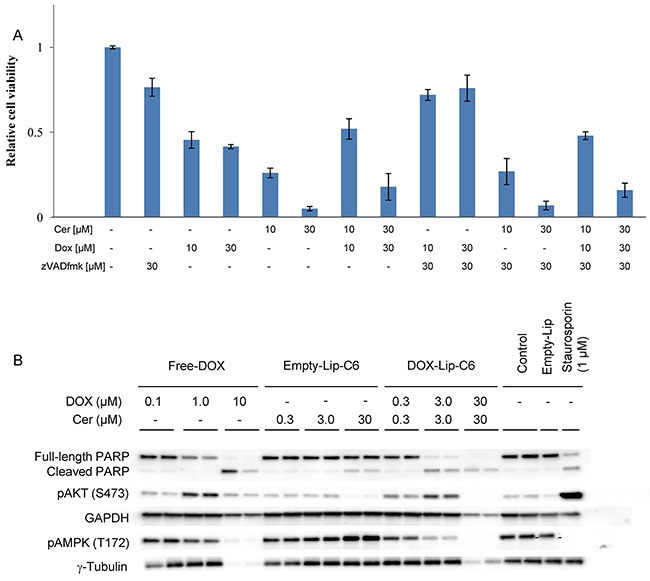
Effects of ceramide and doxorubicin on cell death signaling **(A)** HeLa cells were incubated with various concentrations (1-30 μM) of DOX-loaded liposomes and Free-DOX. Pan-caspase inhibitor zVADfmk (10 or 30 μM) was added to address the effect of caspase-activity on cell viability measured by the MTT assay after 24 h. Bar graphs show mean values from three independent experiments and standard deviations. **(B)** Immunoblotting of HeLa cells were performed to investigate influence of ceramide and DOX on cellular signaling pathways. HeLa cells treated with either Free-DOX (0.1 - 10 μM), Empty-Lip-C6 (0.3 - 30 μM) or DOX-Lip-C6 (0.3 – 30 μM) were lysed, the lysates separated on SDS-PAGE and immunoblotted against PARP, phosphorylated (Ser473) AKT, GAPDH, phosphorylated (Thr172) AMPK and gamma-tubulin in duplicate. Untreated cells, cells treated with Empty-Lip or Staurosporin (1 μM) were used as controls.

The relationship between ceramide and DOX in terms of cell death signaling was assessed by mixing increasing amounts of drug and liposomes with the pan-caspase inhibitor zVADfmk and incubating for 24 h (Figure 6A). The inhibitor partly rescued cells from Free-DOX induced toxicity, but failed to inhibit cell death induced by empty or DOX-loaded ceramide-containing liposomes. DOX-C6-Lip induced PARP cleavage, a hallmark of apoptotic cell death [28], at 3 μM, similarly to Free-DOX, but C6-ceramide alone does so only marginally (Figure 6B). Low concentrations of DOX induced phosphorylation of AKT, whereas this effect was less pronounced at elevated DOX concentrations. In contrast, ceramide does not appear to induce phosphorylation of AKT at these concentrations (10-30 μM), supporting previous findings seen with C6-nanoliposomes *in vitro* [29]. The cytotoxic effect of ceramide could potentially be mediated through AMPK since Empty-C6-Lip enhanced its phosphorylation.

### Ceramide does not enhance the effect of DOX on tumor growth in a mouse model

**Figure 7:**
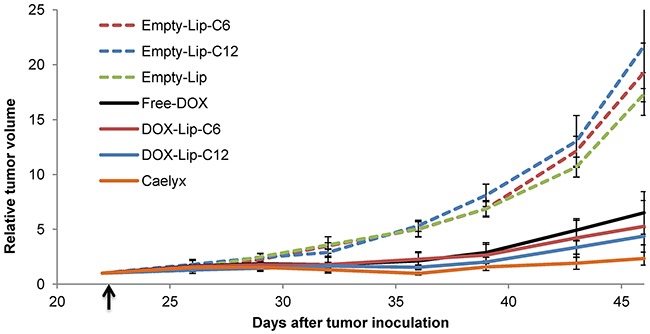
Effect of ceramide liposomes on tumor growth in mice bearing MAS98 12 breast cancer xenografts. The tumor volumes were measured from day 22, i.e. one day prior to injection day (arrow mark) and up to day 47, i.e. 24 days after intravenous injection of DOX-containing liposomes or Free-DOX (8 mg/kg DOX) or a similar amount of empty liposomes. Tumor volumes are shown as relative to the tumor volumes at start of treatment. Data show mean values and standard deviations (n = 7-11 tumors).

The effect of DOX-containing liposomes on tumor growth was studied by intravenous injection of a liposomal formulation corresponding to a DOX dose of 8 mg/kg to mice bearing MAS98.12 patient-derived breast cancer xenografts (Figure 7). Two weeks after treatment all DOX-additions reduced the tumor volume compared to that obtained with the empty liposomes (negative control). Although not statistically significant, ceramide containing liposomes seem to have a slightly better effect on tumor growth than Free-DOX, and Caelyx® seems to have the best effect (Figure 7). The tumor growth was equal for all the empty liposome treatments (Empty-Lip-C6, Empty-Lip-C12 and Empty-Lip), indicating no effect of ceramide alone, regardless of chain length (C6 or C12). Little difference was observed for systemic toxicity between the different DOX-containing liposomes, albeit Free-DOX was more toxic than DOX-Lip-C6 and Caelyx® (Supplementary Figure 4).

## DISCUSSION

### *In vitro*: kinetics and mechanism

A main finding in the present study is that when studying the effect of ceramide inclusion into PEGylated liposomes containing DOX, the toxicity is dependent upon the cell line, the time of incubation and the test assay used. After 24 h incubation ceramide C6 was more effective than ceramide C12 in increasing toxicity to HeLa and HCT116 cells, whereas ceramide C12 was more effective for MDA-MB-231 cells. The MDA-MB-231 cells were less sensitive to the DOX-containing preparations than the two other cell lines after 24 h of incubation as measured with [^3^H]thymidine incorporation. By increasing the incubation time to 72 h the HeLa cells became much more sensitive to all DOX-containing preparations with Free-DOX being the most toxic substance tested (Supplementary Figure 3A/data not shown). When compared to Caelyx®, the ceramide-containing liposomes show higher toxicity at the same DOX-concentrations at incubation time up to 48 h, whereas only minor differences were observed after 72 h, indicating a slower release of DOX from Caelyx®, which has been promoted as a ‘model’ PEGylated liposomal DOX [30]. However, the prolonged circulation time that in most cases is regarded to be favorable, could be the cause of the adverse effects seen for this treatment [31]. We observed an increased cellular toxicity in MDA-MB-231 cells when combining C12 ceramide with Caelyx® (Figure 4) suggesting that inclusion of ceramide in liposomes may be beneficiary. Another phenomenon is the moderate toxic effect of Caelyx® observed to a similar extent in all cell lines when using the MTT assay, indicating that cell energy metabolism is merely slightly disturbed. This is in contrast to the strongly reduced cell proliferation after 72h with Caelyx® in HeLa cells (Figure 2F).

There are several reasons to investigate the potential for using ceramide in combinational drug therapy. For example, pretreatment with ceramide has been shown to resensitize drug-resistant cells to the primary treatment modality comprising DOX- or paclitaxel-loaded nanoparticles [20, 32]. Also, it is known that the synthesis of glycosphingolipids from ceramide is increased in cancer cells [33] thereby counteracting the pro-apoptotic effect of ceramide. By overwhelming the enzymatic processing (saturating GCS) with additional ceramide a more consistent pro-apoptotic signaling may be sustained. However, efficient delivery of drug combinations in the clinical setting depends on a number of factors. Thus, the ability to synchronize the release of the individual therapeutic agents applied in combination, not only allows for simultaneous tumor accumulation, but might also induce a synergistic effect at the intracellular level that could prove to be advantageous.

Assessing the usefulness of adding exogenous ceramide to cells offers some challenges regarding the intracellular metabolism. There are several downstream products of ceramide that have vastly different intracellular effects, such as glycosphingolipids (e.g. gangliosides and cerebrosides), sphingomyelin and sphingosines. Several studies have investigated ceramide C6 as an active ingredient in liposomes, but no studies have compared the effect of ceramide C6 with longer chain-length ceramide species, and as discussed above ceramide C6 and C12 gave different effects on the three cell lines studied. This observation could be explained by cell line (or tissue) specific expression of key enzymes in the glycosphingolipid metabolism, or differences in the lipid composition of the cellular membranes [23]. Moreover, ceramide C12 should be expected to give more stable liposomes [15] and perhaps a slower release of DOX than those containing ceramide C6 [34], a property that might be important to kill slow-growing cells. In agreement with this idea ceramide C12 is more efficient than ceramide C6 on the slow growing MDA-MB-231 cells (doubling time 38 h compared to approximately 24 h for the two other cell lines). Since previous studies with C6-liposomes in these cells show effects in combination with sorafenib [29] or taximofen [35], this result could suggest an even greater effect with C12-liposomes for same or similar drugs in triple negative breast cancer cells.

Our *in vitro* cell toxicity studies revealed that the selected assays resulted in different readout of the cellular toxicity. The cell proliferation assay, measuring incorporation of [^3^H]thymidine, did not reveal any significant effect of ceramide alone after 24 h (Figure 2), while such an effect was evident when using the MTT cell viability assay (Supplementary Figure 3B). Testing the toxic effects on cells after various incubation times may reveal important differences in the cellular response, such as the delay here reported for Caelyx® toxicity. Thus, to understand the mechanisms of added drugs, and especially when trying combinatorial approaches, different types of *in vitro* assays are important.

### *In vivo* studies

The different liposome preparations were intravenously injected in mice with breast cancer xenografts (MAS98.12) to study the effect on tumor growth. These studies showed large effects on the tumor growth of all DOX-containing formulations, but did not show any significant difference between Free-DOX and CER-Lip-DOX. This may be due to insufficient ceramide concentration in the liposomes, since our data do not reveal any effect of ceramide alone, in contrast to previous studies where 20-30x higher final ceramide concentrations were used [36–38]. Fonseca *et al*. suggest that the ratio between ceramide and DOX influences the cytotoxicity of ceramide liposomes [25]. In contrast to their findings, we cannot see any increased toxic effect of a 1:1 ratio *in vivo*, but our data are in agreement with their suggestion of a more efficient ratio of 2 mole ceramide per 1 mole doxorubicin in the liposomal formulation *in vitro*. Increasing the ratio to 10:1 (CER:DOX) did not give an additional effect (Figure 4).

We speculate that the larger effect *in vivo* of Caelyx® compared to our liposomes is due to a greater *in vivo* stability of Caelyx®. If true, different stabilities may be due to the presence of ceramide in our liposomes or the presence of cholesterol in Caelyx®. Although, we did not observe an increased therapeutic effect by adding ceramide to our liposomes, we can of course not exclude the possibility that ceramide might improve the effect in another tumor model, e.g. with a different growth rate.

## MATERIALS AND METHODS

### Materials

Doxorubicin (DOX) was purchased from Eurasia's Chemicals and API (Mumbai, India). All lipids; 1,2-distearoyl-sn-glycero-3-phosphocholine (DSPC), 1,2-distearoyl-sn-glycero-3-phosphoethanolamine-N-[methoxy(polyethylene glycol)-2000] ammonium salt (DSPE-PEG2000), 1,2-dioleyl-sn-glycero-3-phosphoethanolamine (DOPE), N-hexanoyl-D-erythro-sphingosine (Ceramide C6), and N-lauroyl-D-erythro-sphingosine (Ceramide C12), were purchased from Avanti Polar Lipids (Alabaster, AL, USA). DMEM and RPMI 1640 AQmedium, dimethyl sulfoxide (DMSO), ammonium sulfate, sodium chloride (Fluka, Germany), sucrose, Triton X-100, potassium hydroxide, trichloroacetic acid (TCA), formic acid eluent additive for LC-MS (Fluka), acetonitrile LC-MS CHROMOSOLV®, zvAD-FMK, 3-(4,5-dimethyl-2-thiazolyl)-2,5-diphenyl-2H-tetrazolium bromide (MTT; cat#M5655), and penicillin/streptomycin (Pen/Strep P4333) were obtained from Sigma-Aldrich Chemie (Germany). [^3^H]thymidine and Emulsifier Safe scintillation fluid were obtained from Perkin Elmer (USA). Milli-Q water was freshly prepared from the Millipore Milli-Q Biocell water purification system. Caelyx® was bought from Janssen-Cilag International (Belgium). Adrianamycin was purchased from Pfizer, Switzerland. Antibodies against phosphorylated (Thr172) AMPK, phosphorylated (Ser473) AKT and PARP were purchased from Cell Signaling Technologies (USA), anti- GAPDH from Abcam and anti-gamma-tubulin were obtained from Sigma.

### Preparation of liposomes

Liposomes containing DOX and corresponding empty liposomes (Figure 1A) were prepared by the method originally reported by Haran *et al.* [39]. The lipids were dissolved in methanol/chloroform solution (1:1, v/v) and the solvents evaporated on Büchi rotavapor R-124 (Büchi Labortechnik, Flawil, Switzerland) for at least 2 h at 50 mmHg and 65 °C. Lipid films were hydrated by 110 mM ammonium sulfate at 65 °C in a water bath for 1 h. Liposomal suspensions were stored refrigerated at 5 °C overnight and sonicated to desired vesicle size using Ultrasonic processor VC (750 W; Sonics and Materials, Newton, CT, USA). The sonication time (2 min interval) was dependent on the lipid composition and varied from 5 × 2 min to 10 × 2 min. The samples were left to cool for 5 min after each sonication cycle. To form an ammonium sulfate gradient, dialysis was performed for 6 h against 10% (w/v) sucrose [39]. A solution of DOX (22 mg/mL) was added to liposomal suspensions and the loading performed at 65 °C for 1 h. The loading was terminated by the removal of unentrapped DOX by dialysis using tubing with cut off of 12–14 000 Da (Medicell International Ltd., London, UK).

#### Liposome size and size distributions

The size distributions were determined by a photon correlation spectroscopy (Submicron particle sizer model 370, Nicomp, Santa Barbara, CA, USA) as reported previously [40]. The particle intensity was adjusted to approximately 250-350 kHz; the analyses were run in a vesicle mode and expressed as intensity-weight distribution. Three parallels (with a run time of 10 min for each parallel) were determined for each sample measurement.

#### Liposome DOX entrapment efficiency

The unentrapped DOX was separated from DOX-containing liposomes by dialysis, and the DOX content subsequently analyzed by HPLC (Supplementary Table 1). The proportion of DOX present inside the liposome (encapsulated drug) relative to the total amount of drug added to the liposome dispersion was calculated from the amount of DOX present in the liposome samples prior to and after dialysis.

### DOX HPLC-quantification

DOX was quantified using a Waters HPLC system, equipped with a Waters e2795 separations module, a Waters 2489 UV/Visible detector and a C-18 column: XSELECT CSH column XP, 2.5 μm 3.0×75 mm (Waters, Dublin, Ireland). Detection wavelength was 254 nm, and the injection volume was 10 μL. The flow rate was set to 0.5 mL/min and the temperature was set to 25 °C. Two mobile phases were applied for gradient flow condition. Mobile phase A was Milli-Q water with 0.1% (v/v) formic acid and Mobile phase B was acetonitrile with 0.1% (v/v) formic acid. The mixing ratio of Mobile phase A and Mobile phase B was changed linearly from 95:5 to 5:95 (v/v) during 10 min, and with succeeding equilibration sequence of 5 min. Liposome samples and standard samples were prepared in triplicate in Mobile phase A and Triton X-100 95:5 (v/v). Each DOX standard solution (concentration range 5-100 μg/mL) was injected three times into the HPLC (linear standard curves R^2^ value was 0.9997). DOX retention time (RT) was 4.8 min, whereas the Triton X-100 top had a RT of approximately 10.0 min.

### Cell lines

Three commonly used cell lines, routinely tested for mycoplasma, were used in this study. The MDA-MB-231 triple negative breast cancer cell line was cultured in RPMI 1640, while HeLa cervical cancer cells and HCT116 colon carcinoma cells were cultured in DMEM. All the cell lines were obtained from ATCC. Both media were supplemented with 10% (v/v) heat-inactivated fetal calf serum (FCS), 100 U/mL penicillin and 100 μg/mL streptomycin, and maintained at 37 °C in a 5% CO_2_ atmospheric incubator. The experiments started 24 h after cell seeding, to enable attachment of the adherent cells before the corresponding incubation. HeLa and HCT116 cells were seeded to a final concentration of 5000 cells per well in 96-well plates, whereas 8000 cells were seeded for MDA-MB-231 due to a reduced proliferation of these cells. The corresponding number of cells applied in the 24-well plate format was 10 times higher than applied in the 96-well plates.

### *In vitro* cytotoxicity measurements

Different cell lines growing in 24- or 96-well plates were incubated with serial dilutions of Free-DOX, PEGylated liposomal DOX with or without ceramide alone or in combination, at fixed molar ratios, for 24, 48 or 72 h at 37 °C in an atmosphere of 5% CO_2_. The toxicity was assessed either by the commonly used MTT (3-(4,5-dimethylthiazol-2-yl)-2,5-diphenyltetrazolium bromide) cell viability test (Section 2.5.1) or by quantifying [^3^H]thymidine incorporation as a measure for cell proliferation (Section 2.5.2).

#### MTT cell viability assay

Briefly, after end of incubation, the cell medium was aspirated and exchanged with half the volume of medium containing a final concentration of 250 μg/mL MTT. The incubation was continued for 2 h at 37 °C for formation of the formazan-particles, which were dissolved in DMSO with 1% (v/v) NH_4_Cl. The absorbance was read in a plate reader (Biosys Ltd, Essex, UK) at 570 nm, and background from absorbance at 650 nm was subtracted.

#### Cell proliferation measured by [^3^H]thymidine incorporation

To measure DNA synthesis, the cell medium was aspirated, and substituted with serum free cell medium containing [^3^H]thymidine (3 μg/mL; 75 μCi/mL). The incubation was continued for 30 min at 37 °C. The medium was removed and 5% (w/v) TCA was added. After 5 min the cells were washed once with TCA and solubilised with 200 μL of 0.1 M KOH, before mixing with 3 mL scintillation fluid (Perkin Elmer, USA). The radioactivity was counted for 1 min in a scintillation counter (Tri-Carb 2100TR, Packard Bioscience, USA).

### DOX cell extraction

The medium was removed from cells incubated with DOX or DOX-containing liposomes and the cells were then washed twice with PBS before lysis with 0.1% (v/v) Triton X-100, leaving the DOX-loaded liposomes intact. The liposome- and Free-DOX-containing lysates were mixed with 1:1 volume PBS (pH 7.4) and added to a SPE C18 SOLA column (Thermo Fisher Scientific #60109-001) preconditioned by methanol and distilled water. The column containing the mixed lysate was washed once with PBS (pH 7.4) to allow liposomes to pass through the column bed. The column-bound DOX was eluted with methanol including 0.1% (v/v) TFA. The fraction containing liposomal DOX was treated with 1.5% (v/v) Triton X-100 in 5% (v/v) acetonitrile in water to disrupt liposomes and release DOX.

### Western blotting

For immunoblotting of proteins 50,000 cells were incubated for 24 h and subjected to lysis in 50 μL lysis buffer (25 mM Tris-HCl pH 7.6, 150 mM NaCl, 1% (w/v) NP-40, 1% (w/v) sodium deoxycholate, 0.1% (w/v) SDS, 0.2% (w/v) octyl-β-D-glucopyranoside) on ice for 10 min. The resulting protein lysate was mixed 1:1 with 1.5% (w/v) SDS in lysis buffer and sonicated. SDS-PAGE separation was performed on a 4-20% TGX gel (Criterion, Bio-Rad, Oxford, UK) and proteins transferred onto a 0.2 μm PVDF membrane with Transblot Turbo (Bio-Rad) system. Membranes were blocked with 5% (w/v) semi-skimmed milk, incubated with primary antibodies at 4° C overnight, and probed with HRP-conjugated secondary antibodies. The protein bands were visualized by chemiluminescence and measured with Bio-Rad Quantity One on a Chemigenius system.

### Animal studies

All procedures and experiments involving mice were approved by the National Animal Research Authority and were conducted according to the regulations of the Federation of European Laboratory Animal Science Association (FELASA). Mice were kept under pathogen-free conditions, at constant temperature (21.5 ± 0.5°C) and humidity (55 ± 5%); 15 air changes/h and a 12 h light/dark cycle. Distilled water was given *ad libitum*, supplemented with 17-β-estradiol at a concentration of 4 mg/L. All mice used in the experiment were locally bred at the animal facility at Institute for Cancer Research, Oslo University Hospital [41].

The orthotopic basal-like xenograft mice model MAS98.12 has been established by directly grafting human primary breast cancer tissue and serially transplanted, as previously described [42]. For the study, 1-2 mm^3^ pieces of MAS98.12 tumors were implanted bilaterally into the mammary fat pad of female athymic nude *foxn1^nu^* mice (age 6-7 weeks and body weights of 15-20 g). After the tumors reached approximately 5 mm in diameter, the mice were randomly assigned to the different treatment groups (the average volume of each group was 24-30 mm^3^). The empty liposomes (Empty–Lip-C6 and Empty–Lip-C12) were administered as a single intravenous tail vein injection with a dose of 6.5 mg/kg ceramide and with additional 8 mg/kg doxorubicin in the drug loaded liposomes (DOX-Lip-C6 and DOX-Lip-C12. Non-liposomal doxorubicin (Adriamycin®) and PEGylated liposomal doxorubicin (Caelyx®) were injected with 8 mg/kg body weight. Furthermore, saline was used as negative control. Tumor growth was measured twice per week, and tumor volumes were calculated using the formula length × width × width × 0.5.

## CONCLUSION

We have shown that ceramide species with different chain lengths (C6 or C12) induce increased sensitivity of cancer cells to DOX in a cell-specific manner. Furthermore, we have also demonstrated the importance of investigating cell toxicity at different time points and with different cell-based assays to assess the efficacy of drug formulations *in vitro*. Longer incubation times were required to obtain cell toxicity with Caelyx®, the golden standard liposomal marketed product widely used for cancer therapy, compared to our liposomal formulations. Furthermore, our data show that the *in vitro* toxicity analyses of nanocarriers do not necessarily reflect their ability to inhibit tumor growth in mice.

## SUPPLEMENTARY MATERIALS FIGURES AND TABLES



## Abbreviations

CER: ceramide
DOX: doxorubicin
DSPC: distearoyl-sn-glycero-3-phosphocholine
DOPE: dioleoylphosphatidylethanolamine
DSPE: 1,2-distearoylphosphatidylethanolamine
GCS: glucosylceramide synthase
LIP: liposomes
MTT: 3-(4,5-dimethylthiazol-2-yl)-2,5-diphenyltetrazolium bromide
PEG: polyethylene glycol 2000
