# Supplementary material for: Ceramide-containing liposomes with doxorubicin: time and cell-dependent effect of C6 and C12 ceramide
Source: Oncotarget. 2017 Aug 12;8(44):76921–34. doi: 10.18632/oncotarget.20217 (PMC5652752; doi:10.18632/oncotarget.20217)
Supplement: Supplementary file 1 [file oncotarget-08-76921-s001.pdf]

Ceramide-containing liposomes with doxorubicin: time and cell-dependent effect of C6 and C12 ceramide

SUPPLEMENTARY MATERIALS

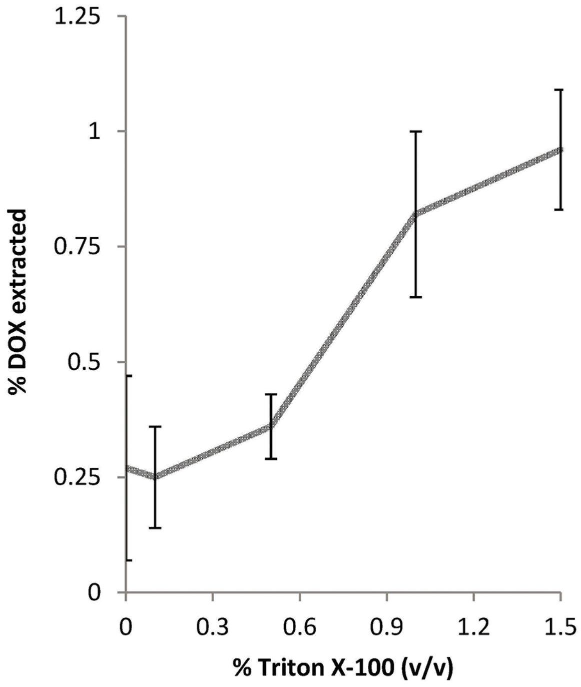

Supplementary Figure 1: Determination of Triton X-100 sufficient for liposomal lysis *in vitro*. Increasing concentration of detergent (0-1.5% (v/v)) Triton X-100 was given to liposomes for 1 mins and the amount of DOX released was quantified with HPLC. Data show mean values and standard deviations (n = 3).

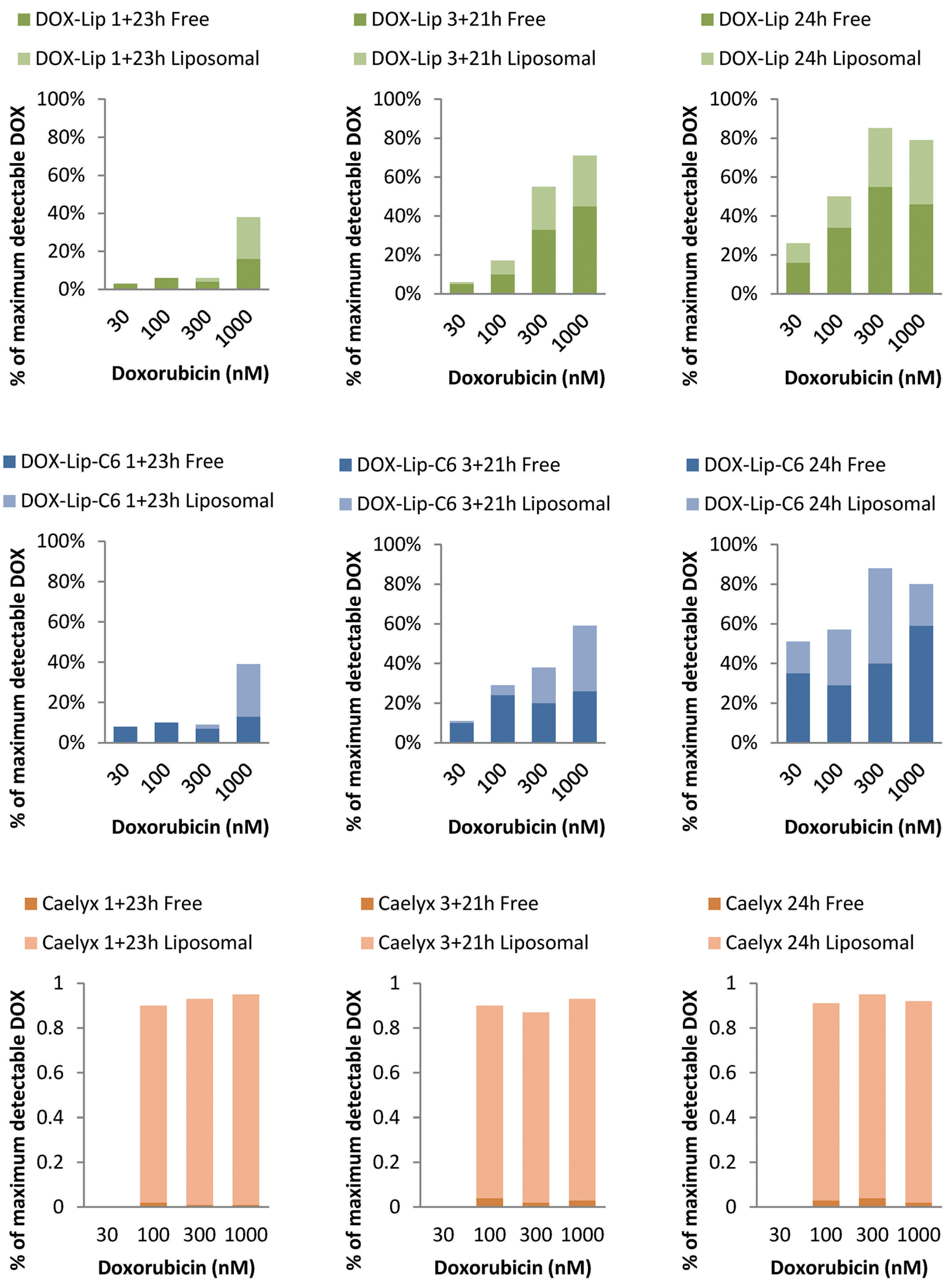

Supplementary Figure 2: Effect of ceramide on doxorubicin release from liposomes. HeLa cells were incubated with various concentrations (30 – 1000 nM) of four different DOX-loaded liposomes and free DOX. The cell medium was removed after 1 or 3 h; the cells were washed and further incubated in medium for 23 or 21 h, correspondingly. Following the complete incubation time of 24 h the cell medium was removed, cells washed and lysed with 0.1% (v/v) Triton X-100. DOX still encapsulated in liposomes was separated from free/released DOX by solid phase extraction and quantified. The data show the percent of DOX being contained in liposomes or released from liposomes. Mean values obtained by analyzing 3 replicates are shown.

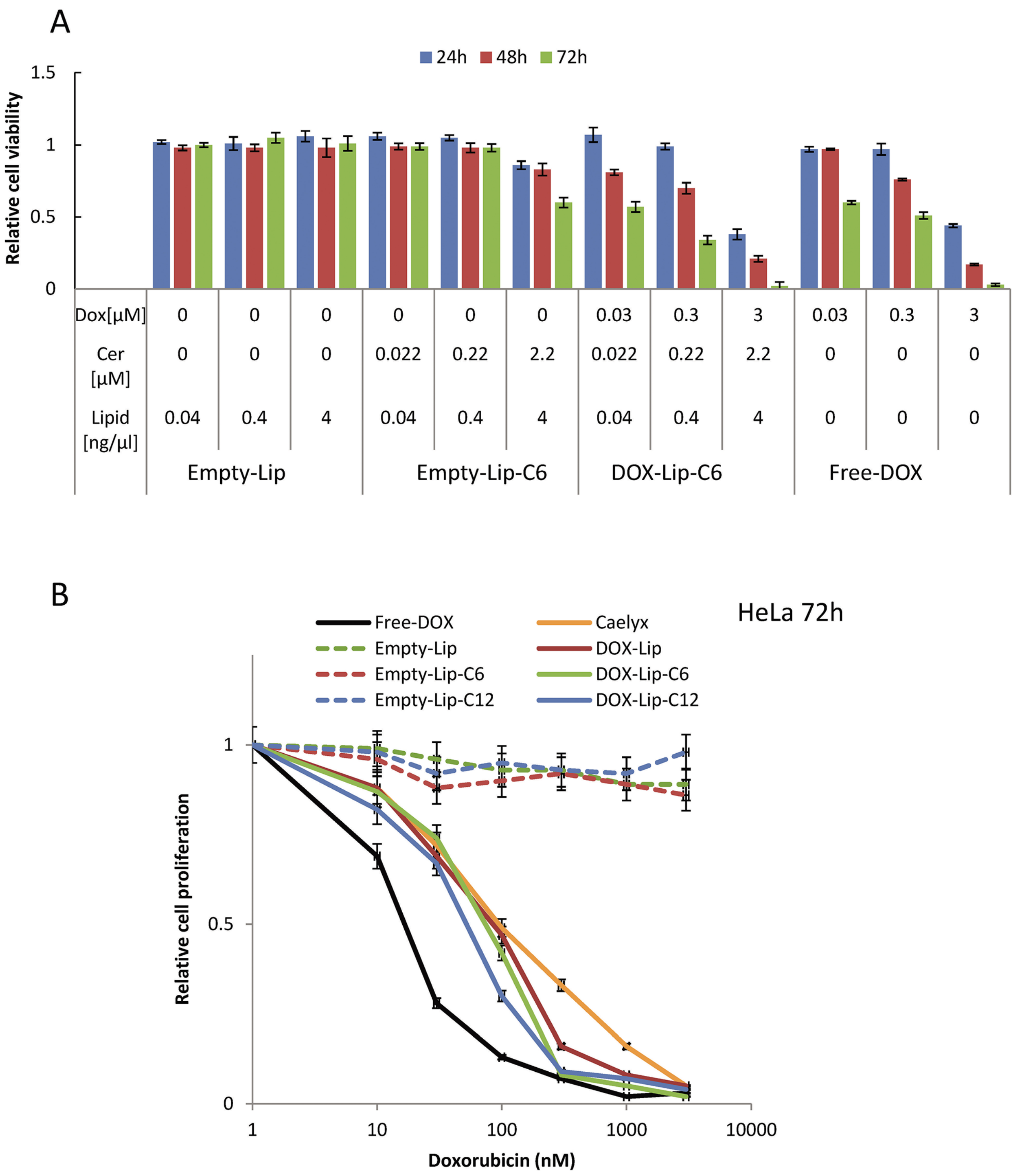

Supplementary Figure 3: (A) Dose-dependent effect of liposomal doxorubicin on cell proliferation. HeLa cells were incubated for 72 h with various concentrations of liposomal DOX (10-3000 nM) followed by measuring incorporation of [^{3}H]thymidine. Free-DOX, empty liposomes with the same lipid concentration or no treatment was used for comparison. **(B)** Dose-dependent effect of liposomal doxorubicin on cell viability. HeLa cells were incubated (24, 48 and 72 h) with various concentrations of DOX (0 - 3000 nM) incorporated into ceramide C6 liposomes and compared to Free-DOX and empty liposomes. The cell viability was determined by the MTT assay. The data show the mean values from at least three independent experiments and standard deviations.

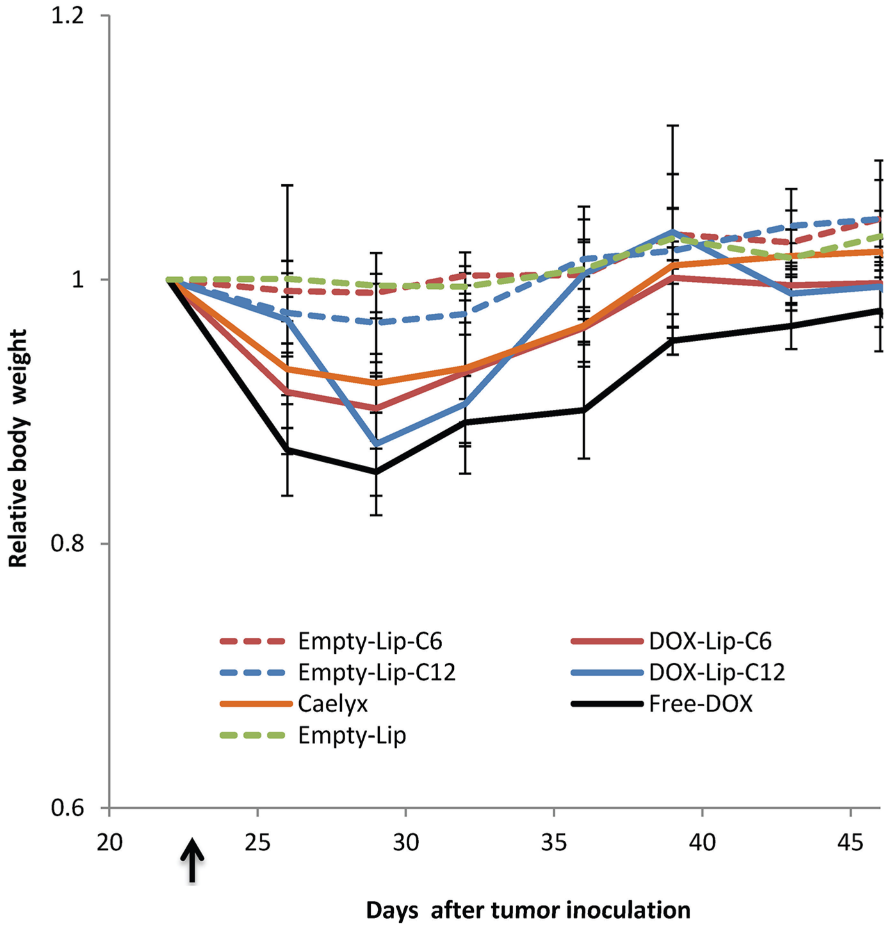

Supplementary Figure 4: Tolerance of injected substances in mice bearing MAS98. 12 breast cancer xenografts measured as body mass relative to the initial weight. The body masses were measured up to day 24 after intravenous injection of DOX-containing liposomes or Free-DOX (8 mg/kg DOX) or a similar amount of empty liposomes. Data show mean values and standard deviations (n = 4-6). Arrow indicates time point for injection, 23 days after inoculation.

Supplementary Table 1: Characterization of the liposomes we made for the present study

Liposome type	DOX entrapment efficacy (%) ± SD	Size distribution		Zeta potential (mV)
		Mean diameter (nm) ± SD	PDI	
DOX-Lip-C6	94.5 ± 1.8	119 ± 54	0.209	n.d.
Empty-Lip-C6	n.a.	118 ± 50	0.209	-8.20 ± 0.64
DOX-Lip-C12	95.2 ± 2.0	127 ± 55	0.228	n.d.
Empty-Lip-C12	n.a.	124 ± 49	0.228	-8.60 ± 0.65
DOX-Lip	93.1 ± 1.2	122 ± 55	0.208	n.d.
Empty-Lip	n.a.	114 ± 53	0.223	-3.50 ± 0.27
Caelyx®	*n.d.*	92 ± 12	0.091	-2.70 ± 0.12

n.a.: not applicable, n.d.: not done.

The unentrapped DOX was separated from DOX-containing liposomes by dialysis, and the DOX content subsequently analyzed by HPLC. The proportion of DOX present inside the liposome (encapsulated drug) relative to the total amount of drug added to the liposome dispersion was calculated form the amount of DOX present in the liposome samples prior and after dialysis:

$$\frac{\text{Amount of DOX}_{\text{pre-dialysis}}}{\text{Amount of DOX}_{\text{post-dialysis}}} \times 100\% = \text{ Liposomal DOX entrapment (\%)}$$

Size measurements were performed over 10 mins with photon correlation spectroscopy in triplicates (see Methods) and mean diameter recorded with standard deviations. Polydispersity index (PDI) calculated as per manufacturer's instructions.

Supplementary Table 2: IC50 values calculated for liposomal doxorubicin treatment in the three cell lines tested

Cell proliferation	IC50 (nM) ± SD				
**Cell line - time point**	**DOX-Lip-C6**	**DOX-Lip-C12**	**DOX-Lip**	**Free-DOX**	**Caelyx®**
HeLa - 24h	96 ± 15	145 ± 18	185 ± 23	151 ± 12	>3000
HCT116 - 24h	55 ± 6	66 ± 3	72 ± 10	74 ± 10	>3000
MDA-MB-231 - 24h	670 ± 20	447 ± 13	630 ± 29	668 ± 33	>3000
**Cell viability**	**IC50 (nM) ± SD**				
**Cell line - time point**	**DOX-Lip-C6**	**DOX-Lip-C12**	**DOX-Lip**	**Free-DOX**	**Caelyx®**
HeLa - 24h	102 ± 28	985 ± 13	210 ± 21	296 ± 16	>10000
HCT116 - 24h	294 ± 18	642 ± 32	340 ± 22	465 ± 41	>10000
MDA-MB-231 - 24h	264 ± 12	107 ± 8	182 ± 16	304 ± 23	4020 ± 16

50% inhibitory concentrations (IC50) were determined for liposomal and non-liposomal doxorubicin-formulations in three cell lines (HeLa, HCT116, MDA-MB-231) for cell proliferation by [3H]thymidine incorporation assay and cell viability by MTT assay.
